# Remarkably efficient removal of toxic bromate from drinking water with a porphyrin–viologen covalent organic framework†

**DOI:** 10.1039/c9sc04663a

**Published:** 2019-11-22

**Authors:** Tina Skorjanc, Dinesh Shetty, Felipe Gándara, Liaqat Ali, Jesus Raya, Gobinda Das, Mark A. Olson, Ali Trabolsi

**Affiliations:** Science Division, New York University Abu Dhabi Saadiyat Island Abu Dhabi United Arab Emirates ali.trabolsi@nyu.edu; Department of Chemistry, Khalifa University P.O. Box: 127788 Abu Dhabi United Arab Emirates; The Materials Science Factory, Instituto de Ciencia de Materiales de Madrid–CSIC, 28049 Sor Juana Inés de la Cruz 3 Madrid Spain; Core Technology Platform, New York University Abu Dhabi Saadiyat Island Abu Dhabi United Arab Emirates; Membrane Biophysics and NMR, Institute of Chemistry, UMR 7177, University of Strasbourg, CNRS Strasbourg France; School of Pharmaceutical Science and Technology, Tianjin University 92 Weijin Rd., Nankai District Tianjin P. R. China

## Abstract

The presence of carcinogenic bromate (BrO_3_^−^) in drinking water became a global concern and efforts towards its removal mainly focused on addressing the source. Herein, we rationally designed a porphyrin-based covalent organic framework (**PV-COF**) with a cationic surface to provide electrostatic interactions and a porphyrin core to induce hydrogen bonding interactions for the efficient removal of BrO_3_^−^ from water. Through H-bonding and electrostatic interactions, **PV-COF** exhibited an exceptional bromate removal efficiency (maximum adsorption capacity, *Q*_max_: 203.8 mg g^−1^) with the fastest uptake rate (*k*_ads_) of 191.45 g mg^−1^ min^−1^. The bromate concentration was reduced to far below the allowed concentration in drinking water (10 ppb) within 20 minutes. We studied the relationship between bromate adsorption and COF surface modification by metalation of the porphyrinic core or neutralization of the viologen linkers by chemical reduction. The bromate adsorption mechanism was studied by EDAX mapping and molecular simulations, and it was found that ion exchange and hydrogen bonding formation drive the adsorption. Importantly, **PV-COF** could be easily recycled several times without compromising its adsorption efficiency.

## Abstract 2

A cationic COF removes carcinogenic bromate with a remarkable rate constant of 191.45 g mg^−1^ min^−1^.

## Graphical abstract

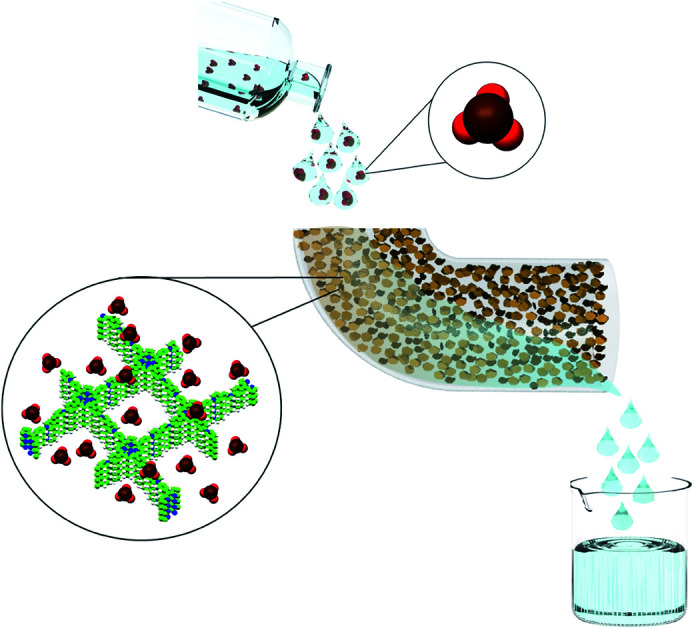

## Introduction

Bromate (BrO_3_^−^) is a toxic substance responsible for the recall of large quantities of bottled drinking water in the US, Europe and across the Middle East.^1–4^ It is formed in drinking water during the processes of ozonolysis and electrolysis of both fresh and seawater.^5^ On account of its serious toxic effects, which include central nervous system depression, haemolytic anemia, pulmonary edema, and even cancer,^6^ both the World Health Organization (WHO) and the Environmental Protection Agency (EPA) have set the maximum contaminant level of bromate in drinking water at 10 ppb.^7^ Ozone treatment is one of the most common methods used to disinfect drinking water and as a result can lead to significant levels of bromate ingestion ranging from 120 to 180 μg per day, much higher than is safely allowed.^8^ In consideration of the difficulties in removing bromate from water, efforts on reducing bromate levels of drinking water have mostly been limited to treating its source: by removal of its precursors (Br^−^) before ozonolysis and/or control of the ozonolysis process in order to minimize BrO_3_^−^ formation. Unfortunately, these processes require water treatment plants to make complex impractical adjustments in their day-to-day operations, and thus the water treatment industry is searching for accessible ways to remove bromate post-ozonolysis. This is an important but largely understudied area in drinking water research.^9^ Among the reported post-treatment methods (physico-chemical, electrochemical or light-triggered reduction, bio-reduction, and various forms of dialysis),^10^ adsorption is the most scalable and cost-effective method whereby activated carbons^11–13^ and inorganic substances^6,14^ have predominantly been used as BrO_3_^−^ sorbents. Yet, these materials suffer from low efficiency, limited and cost-ineffective regeneration ability, and slow kinetics. In contrast, organic polymers comprised of lightweight elements are known to have high water stability and excellent pollutant uptake capacities and can be easily regenerated, but have not been investigated as bromate sorbents.

Covalent organic frameworks (COFs) are a novel class of porous, crystalline materials with tunable structures and extensive surface areas.^15,16^ COFs have been utilized for the removal of various pollutants from water, including toxic dyes,^17^ heavy metals,^18–20^ pharmaceuticals,^21^ and other toxins.^22^ However, adsorption of BrO_3_^−^ is unprecedented for COFs. Herein, we report a cationic porphyrin COF with viologen units (**PV-COF**) obtained through the Zincke reaction^23^ for the efficient removal of bromate from water. While a handful of porphyrin-based COFs have been reported,^24–26^ they are synthesized through the formation of boronic esters or imine bonds, which are known to have limited chemical stability. Contradistinctively, **PV-COF** is formed from the generation of a quaternary ammonium salt, which does not easily hydrolyze.^27^ We tested the utility of **PV-COF** in removing bromate from drinking water because of its inherent properties, whereby its cationic charge can induce favorable electrostatic interactions with anionic BrO_3_^−^, and its porphyrin core can form hydrogen bonds^28^ with BrO_3_^−^. Bromate adsorption by **PV-COF** was extremely fast with a remarkable uptake rate constant of 191.45 g mg^−1^ min^−1^. At this rate, the reduction of an aqueous bromate concentration of 50 μg L^−1^ to less than 3 μg L^−1^, which is far below the allowed concentration in drinking water, occurred in just 20 minutes. The material exhibited a maximum uptake capacity (*Q*_max_) of 203.8 mg g^−1^, which is one of the highest values reported to date. In addition, we post-synthetically modified **PV-COF** through metallation and chemical reduction, and tested the ability of products to adsorb BrO_3_^−^ in order to understand the structure–property relationships which governed their performance.

## Results and discussion

**Fig. 1:**
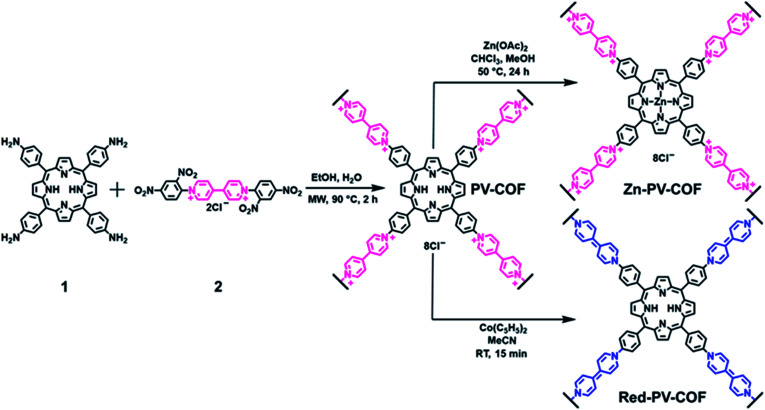
Synthetic route to **PV-COF** through the Zincke reaction and its post-synthetic modifications by metalation of the porphyrin core (**Zn-PV-COF**)and chemical reduction of viologen units from a cationic to a neutral form (**Red-PV-COF**).

**Fig. 2:**
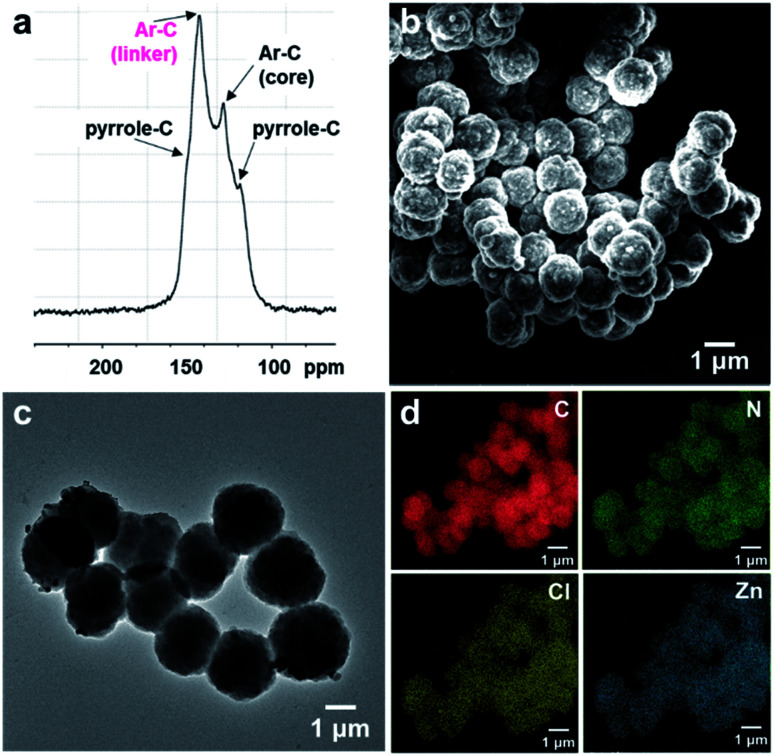
(a) ^13^C solid-state NMR spectrum of **PV-COF** with peaks assigned to relevant carbon atoms; (b) SEM image of **PV-COF** shows spherical particles with an average size of 1.4 μm; (c) TEM image of **PV-COF**; (d) elemental mapping of **Zn-PV-COF** showing an even distribution of constituent elements (C, N, Cl and Zn).

**PV-COF** was synthesized through the Zincke reaction between an amino-derivative of porphyrin (**1**) and the Zincke salt (**2**). In a typical experiment, **1** and **2** were reacted in a 1 : 2 molar ratio in a 4 : 1 mixture of ethanol : water under microwave irradiation at 90 °C for 2 hours (Fig. 1). The crude solid was purified by extensive washing with ethanol and chloroform, and **PV-COF** was obtained in high yield (80 mg COF from 50 mg starting porphyrin) as a powder insoluble in various solvents (water, ethanol, THF, chloroform, and DMSO). The Fourier transform infrared (FT-IR) spectrum of **PV-COF** showed signals corresponding to –C=N– stretching at ∼1603 cm^−1^ and 1630 cm^−1^ belonging to the porphyrin and bipyridinium subunits, respectively (Fig. S1†). In addition, signals for –N–O bond vibrations at 1340 and 1530 cm^−1^ that are present in the FT-IR spectrum of **2** are absent in the spectrum of **PV-COF**, which confirms the success of the Zincke reaction. Molecular-level characterization of **PV-COF** was further achieved using ^13^C cross-polarization magic angle spinning (CP-MAS) solid state NMR (Fig. 2a), which exhibits a strong peak at 142.56 ppm that corresponds to the pyridyl carbon atoms (Ar–C linker) and a peak at 128.39 ppm that corresponds to phenyl carbons of the porphyrin subunit (Ar–C core). We also observed medium intensity peaks at 123.65 and 149.25 ppm that correspond to the pyrrole rings of the porphyrin moiety. These findings confirmed the formation of the network structure as shown in Fig. 1.

Morphological studies on **PV-COF** using scanning electron microscopy (SEM) and transmission electron microscopy (TEM) revealed the presence of uniform spherical particles (Fig. 2b and c). Elemental mapping using energy dispersive spectroscopy (EDS) showed an even distribution of relevant elements (C, N and Cl) throughout the spherical particles (Fig. S2†). An average diameter of 1.4 μm ± 0.18 μm for these spheres was calculated from TEM micrographs (Fig. S3a†). Similarly, dynamic light scattering measurements provided an average diameter of 1.8 μm ± 0.2 μm with a polydispersity index of 0.206 (Fig. S3b†). The surface charge of the PV-COF particles was found to be positive (*ζ*-potential = +13.6 mV) owing to the presence of viologen units in their backbone (Fig. S4†). The porosity of **PV-COF** was characterized by nitrogen gas sorption isotherm measurements. A modest Brunauer–Emmett–Teller (BET) surface area of 38.2 m^2^ g^−1^ (Fig. S5a†) was calculated for the material, possibly due to the presence of chloride counterions that may block some of the network pores. The network was mesoporous in nature with a pore size of 2.3 nm (Fig. S5b†), which is in good agreement with the material's calculated pore size. Thermogravimetric analysis of **PV-COF** confirmed that ∼80% of the material remained stable up to ∼400 °C (Fig. S6†).

**Fig. 3:**
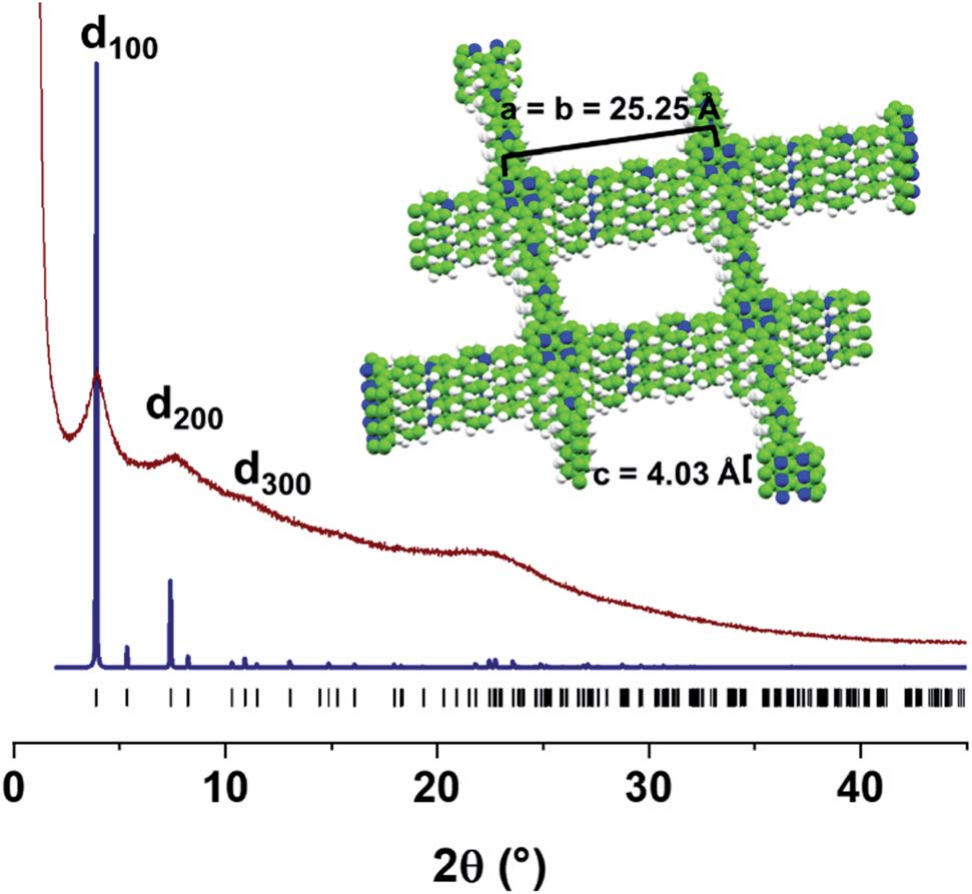
Experimental PXRD pattern of the as-synthesized **PV-COF** (brown line) compared with the simulated pattern using the optimized crystal model (blue line), built in the *P*4̄ unit cell with *a* = *b* = 25.25 Å, and *c* = 4.03 Å. Inset: space-filling view of stacked layers along *x* and *y*-axes.

According to its powder X-ray diffraction (PXRD) pattern, **PV-COF** is crystalline with Bragg diffraction peaks at 2*θ* = 3.86°, 7.39°, and 10.90°. They were assigned to the (100), (200), and (300) Miller planes, respectively, in agreement with a tetragonal unit cell (Table S1†). Thus, a crystal model was built and geometrically optimized in the tetragonal *P*4̄ space group, where **PV-COF** forms square layers that are disposed parallel to the *ab* plane, with an optimized lattice parameter of *a* = 25.25 Å. In the fully eclipsed configuration, the **PV-COF** layers are 4.03 Å apart along the *c* axis, according to the optimized model and corresponding to the broad peak centered at 2*θ* = 22.4° in the experimental PXRD pattern. As shown in Fig. 3, the simulated PXRD pattern of this model is in good agreement with the experimental data. Both the crystallinity and morphology were unaffected by acidic and basic pH (Fig. S7 and S8†).

**Fig. 4:**
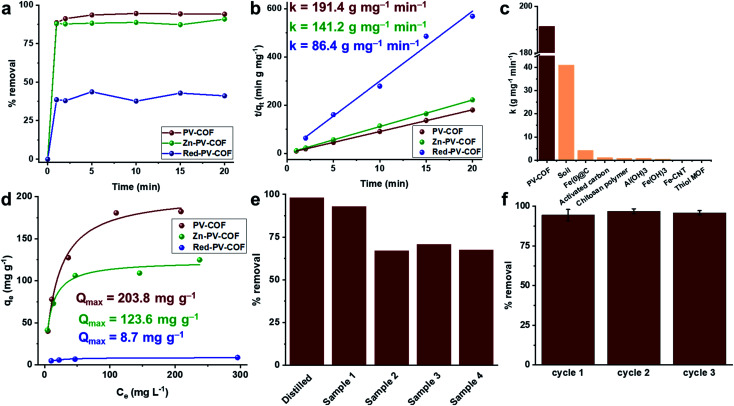
Bromate adsorption results. (a) % removal of BrO_3_^−^ by **PV-COF**, **Zn-PV-COF** and **Red-PV-COF** with 50 μg L^−1^ initial concentration of BrO_3_^−^ at different time points (*n* = 3); (b) the pseudo-second-order kinetic plots for BrO_3_^−^ adsorption by **PV-COF**, **Zn-PV-COF** and **Red-PV-COF** along with associated rate constants (*k*); (c) a comparison of the rate constants of **PV-COF** and other reported bromate adsorbents; (d) Langmuir non-linear isotherm model fitting for **PV-COF**, **Zn-PV-COF** and **Red-PV-COF** along with determined maximum adsorption capacities (*Q*_max_) at BrO_3_^−^ concentrations in the range 12.5–200 mg L^−1^; (e) % removal of BrO_3_^−1^ after 20 min incubation of **PV-COF** with commercial water samples from the UAE, Italy and Norway to which BrO_3_^−1^ was added at a concentration of 50 μg L^−1^. Water samples contained competitive anions, including bicarbonate (0–182 mg L^−1^), sulfate (0–86 mg L^−1^), chloride (1.3–77 mg L^−1^), nitrate (0.3–1.1 mg L^−1^) and fluoride (0.1–0.5 mg L^−1^); (f) regeneration efficiency of **PV-COF** for BrO_3_^−^ adsorption; uptake efficiency is preserved for at least three cycles.

Porosity, hydrothermal stability, cationic surface, and the ability to form both electrostatic and hydrogen bonds with oxoanions encouraged us to investigate the BrO_3_^−^ adsorption capability of **PV-COF**. To mimic practical conditions in a full-scale drinking water plant, the starting BrO_3_^−^ concentration was set at 50 μg L^−1^.^29^ In a typical experiment, **PV-COF** (5 mg) was incubated with a NaBrO_3_ solution (10 mL) and 1 mL aliquots were removed at different time points (1, 2, 5, 10, 15, and 20 min). These fractions were passed through a syringe filter (0.2 μm pore size) and residual solutions were analyzed with an HPLC coupled to a triple quadrupole mass spectrometer (HPLC-QqQMS) to quantify the amount of BrO_3_^−^ (details in the ESI†). We found a remarkable decrease in the stock concentration of BrO_3_^−^ to less than 3 μg L^−1^ within 20 minutes of **PV-COF** treatment, which corresponded to over 95% removal (Fig. 4a). The rate constant for the adsorption process was determined by fitting the data to a pseudo-second order kinetic model^30,31^ and an adsorption rate of 191.45 g mg^−1^ min^−1^ was calculated with a correlation coefficient of ∼1, which is a value higher than that of any other reported adsorbent (Fig. 4b, c and Table S2†). The maximum BrO_3_^−^ absorption capacity of **PV-COF** was estimated by an isotherm study using a range of bromate concentrations (12.5 to 200 mg L^−1^). We fitted the adsorption data to the Langmuir and Freundlich non-linear adsorption models,^32,33^ but a better fit was obtained with the former (*R*^2^ = 0.98 and 0.92, respectively). A *Q*_max_ of 203.8 mg g^−1^ was calculated (Fig. 4d and S9†), which is among the highest values reported to date for any class of BrO_3_^−^ sorbents (Table S2†). The mechanism of BrO_3_^−^ adsorption involves exchange of counter ions. The relative amounts of Cl and Br change from 32.9% and 0.0%, respectively, prior to adsorption (Fig. S2†) to 4.6% and 18.8%, respectively, post adsorption (Fig. S10†). This reduction in the relative amount of Cl and the increase in the amount of Br strongly suggest that Cl^−^ counter ions are replaced by BrO_3_^−^. As a result of BrO_3_^−^ adsorption, the total surface area of the material decreases to 30.0 m^2^ g^−1^ (Fig. S11†), the average particle diameter in solution increases to 2.2 μm because of swelling and the average surface charge decreases to +5.2 mV as a result of surface-adsorbed BrO_3_^−^ (Fig. S12†).

To mimic the real-life conditions of adsorption, we performed experiments with commercial water samples to which NaBrO_3_ was added at 50 μg L^−1^. These samples contained other anions, typically in thousand times higher concentration ranges, including bicarbonate (0–182 mg L^−1^), sulfate (0–86 mg L^−1^), chloride (1.3–77 mg L^−1^), nitrate (0.3–1.1 mg L^−1^) and fluoride (0.1–0.5 mg L^−1^). In spite of the presence of competitive anionic species at high concentrations, **PV-COF** removed up to 93% of BrO_3_^−^ in 20 minutes (Fig. 4e). The effect of pH on the adsorption was also tested and it was found that an acidic pH of 5 does not alter % removal in the first 20 minutes, but a basic pH of 8 decreases it to ∼70% (Fig. S13†). This can be explained by a partial loss of the cationic character of **PV-COF**: it is well known that bases reduce viologen units to radical cations,^34^ which have a lower affinity for bromate than dicationic viologens.

To evaluate the role that the network core units and surface modifications play in impacting BrO_3_^−^ removal efficiency, we chemically modified the surface of **PV-COF** post-synthetically by: (1) metallating the porphyrin units with zinc metal ions (**Zn-PV-COF**, Fig. 1) which could potentially coordinate to bromate, or (2) chemically reducing the bipyridinium subunits using cobaltocene to neutralize the COF surface (**Red-PV-COF**, Fig. 1). Neither of the two modifications resulted in a change in the morphology as evidenced by SEM and TEM imaging (Fig. S14 and S15†). The average size of the particles remained the same for **Zn-PV-COF** (∼1.4 μm), whereas there was a slight decrease in the size for **Red-PV-COF** (∼1.2 μm), likely because of the loss of counterions (Fig. S16a and c†). EDS mapping confirmed an even distribution of the transition metal as well as other constituent elements throughout **Zn-PV-COF** (Fig. 2d). The *ζ*-potential of the metallated COF was measured to be +25.2 mV owing to the positively charged metal centers introduced (Fig. S16b†). Conversely, the *ζ*-potential for **Red-PV-COF** became negative and was measured to be −24.1 mV (Fig. S16d†). FT-IR spectra of **Zn-PV-COF** and **Red-PV-COF** showed –C=N– vibration signals from the porphyrin subunit at 1603 cm^−1^, but the latter lacked a signal at 1630 cm^−1^ due to the loss of –C=N– bonds following reduction of the bipyridinium units (Fig. S17†). In addition, **Zn-PV-COF** exhibited a strong signal at 1001 cm^−1^, shifted from 966 cm^−1^ in **PV-COF**. This indicated an interaction of the –C–N– bonds with the Zn metal center. Porosity and pore size distributions remained similar after metalation (Fig. S5c and d†), but reduction resulted in a significant increase in the surface area of **Red-PV-COF** to 306 m^2^ g^−1^ (Fig. S5e and f†). This can be explained by the removal of counter ions, which block some of the pores in **PV-COF**. Neither of chemical modifications imparted significant radical character to the material as evidenced by electron paramagnetic resonance (EPR) spectra (Fig. S18†). The PXRD pattern of **Zn-PV-COF** and **Red-PV-COF** showed a broadening of the peak corresponding to the (200) plane with increased intensity and a reduced intensity of the peak corresponding to the (100) plane (Fig. S19†).

Having fully characterized the modified materials, BrO_3_^−^ removal experiments were conducted as previously described. Adsorption rates were found to be 141.17 g mg^−1^ min^−1^ and 86.43 g mg^−1^ min^−1^ for **Zn-PV-COF** and **Red-PV-COF**, respectively (Fig. 4b). We hypothesized that the introduction of Zn atoms into the porphyrin cores may prevent the formation of hydrogen bonding between the core and BrO_3_^−^, which may effectively reduce the rate of adsorption. A molecular simulation of the interaction between BrO_3_^−^ anions and the central porphyrin units indicated that the bromate anions might be forming H-bonds with the central porphyrin core (Fig. S20†). On the other hand, when considering the periodic **PV-COF** structure, the results of simulation on the BrO_3_^−^ sorption sites indicated that bromate anions are primarily adsorbed by forming H-bonds with the hydrogen atoms of the porphyrin pyrrole groups that are pointing into the pores (Fig. S20†). This was experimentally confirmed by FT-IR analysis, where the –N–H vibrations at ∼3100 cm^−1^ almost disappear post-bromate adsorption (Fig. S21†). Although Zn could serve as a coordination center for anions, it also increased the molecular weight of the material, so the net effect was a reduction in the BrO_3_^−^ adsorption rate. Similarly, neutralization of the bipyridinium subunit's cationic charge prevented the formation of electrostatic ion-paired interactions between the sorbent and BrO_3_^−^, the net effect of which reduced the adsorption rate even more dramatically. These results suggest that electrostatic interactions play a major role in the adsorption process. In terms of maximum uptake capacity for BrO_3_^−^, **Zn-PV-COF** and **Red-PV-COF** do not perform as extraordinarily as **PV-COF**, but **Zn-PV-COF** nevertheless has one of the highest reported *Q*_max_ values at 123.60 mg g^−1^ (Fig. 4d).

Finally, we tested the regeneration ability of the best-performing **PV-COF**. Adsorbed BrO_3_^−^ could be desorbed by simple washing with a 40 mM solution of NaOH followed by neutralization with 10 mM HCl, as previously reported (details in the ESI†).^35^ We tested our material for three consecutive cycles without significant loss of adsorption efficiency at both lower (50 μg L^−1^) and higher (50 mg L^−1^) BrO_3_^−^ concentrations (Fig. 4f and S22†). Furthermore, **PV-COF** preserved its morphology after the three cycles of adsorption (Fig. S23†). Additionally, no significant changes in the fingerprint region of the FT-IR spectrum of the material were observed (Fig. S24†), and crystalline nature was preserved (Fig. S25†). Because remediation of ground and drinking water typically involves packed-bed columns,^36^ we also conducted adsorption and regeneration experiments in the continuous flow setup (details in the ESI, Fig. S26†). The concentration of BrO_3_^−^ was decreased from 50 μg L^−1^ to below the detection limit for three consecutive cycles of adsorption.

## Conclusions

In conclusion, we designed and synthesized a novel porphyrin-based crystalline material, **PV-COF**, through the Zincke reaction, which proceeded in a very high yield. **PV-COF** exhibited a high efficiency for the adsorption of bromate, a carcinogen introduced into water during the process of ozonolysis. The rate of adsorption was measured to be 191.45 g mg^−1^ min^−1^, which is one of the fastest rates reported to date, with a maximum uptake capacity value of 203.8 mg g^−1^. Chemical modifications to the structure of **PV-COF**, such as neutralization of its positive charge and introduction of a metal center, both reduced the uptake of BrO_3_^−^. These data indicated that electrostatic interactions and hydrogen bonding both contributed largely to the remarkable BrO_3_^−^ uptake ability of this material. This is the first report on the use of COFs for the removal of toxic bromate from water, which will likely generate interest in the community for the continued design of COF-based materials for applications in environmental remediation.

## Conflicts of interest

There are no conflicts to declare.

## Supplementary Material

SC-011-C9SC04663A-s001

SC-011-C9SC04663A-s002
