# Supplementary material for: Remarkably efficient removal of toxic bromate from drinking water with a porphyrin–viologen covalent organic framework
Source: Chem Sci. 2019 Nov 22;11(3):845–50. doi: 10.1039/c9sc04663a (PMC8145354; doi:10.1039/c9sc04663a)
Supplement: SC-011-C9SC04663A-s002 [file SC-011-C9SC04663A-s002.pdf]

Electronic Supplementary Material (ESI) for Chemical Science.
This journal is © The Royal Society of Chemistry 2019

Supporting Information

Remarkably efficient removal of toxic bromate from drinking water by porphyrin-viologen covalent organic framework

*Tina Skorjanc, Dinesh Shetty, Felipe Gándara, Liaqat Ali, Jesus Raya, Gobinda Das, Mark Anthony Olson, and Ali Trabolsi**

Materials characterization

FTIR studies were carried out on Agilent 670 IR spectrometer in the attenuated total reflectance (ATR) mode. TGA experiments were performed on a TA SDT Q600 with a heating rate of 10 °C min^{-1} over a temperature range of 25–1000 °C. Powder X-ray diffraction (PXRD) measurements were carried out on Bruker D8 Advance X-ray diffractometer with Cu K_α (λ = 1.5405 Å) radiation source operating at 40 kV and 30 mA. The patterns were recorded with divergent slit of 1/16° over the 2θ range of 1–50° with step size = 0.01°. Surface area measurements were conducted on a Micromeritics 3Flex gas sorption analyzer. Samples (~30 mg) were degassed at 85 °C for 20 h and then backfilled with N_2. Adsorption isotherms were generated by incremental exposure to ultrahigh-purity nitrogen up to 1 atm in a liquid nitrogen bath, and surface parameters were determined using BET adsorption models included in the instrument software (Micromeritics ASAP 2020 V4.00). SEM images were obtained from FEI Quanta 450FEG. TEM images were obtained from a FEI-Titan 300 operating at 200 kV. Dynamic light scattering (DLS) measurements were performed on a Malvern Zetasizer NanoSeries. EPR spectra were recorded on CMS 8400 EPR spectrometer with a centerfield at 337 mT, sweep width of 20 mT, and power attenuation of 20 dB, and visualized with EPR Commander 6.0.

1,1'-bis(2,4-dinitrophenyl)-[4,4'-bipyridine]-1,1'-diium dichloride (tetranitroviologen, **TNV**, **2**) was synthesized by refluxing 4,4'-bipyridine (4 g, 25.60 mmol) and 1-chloro-2,4-dinitrobenzene (26 g, 89.60 mmol) in 150 mL of anhydrous acetonitrile under Ar for 72 h. After the reaction was complete, the mixture was filtered, and the solid was washed with acetonitrile (50 ml, once) and diethyl ether (40 ml, four times, followed by soaking). Pure **TNV** was obtained in 75% yield. ^{1}H NMR (400 MHz, MeOD δ): δ 9.46 (d, 4H), 9.39 (d, 2H), 8.90–8.94 (m, 6H), 8.29 (d, 2H) ppm. ^{13}C NMR (126 MHz, D_2O) δ 152.66, 149.89, 146.86, 142.82, 138.24, 131.10, 130.75, 127.55, 122.82 ppm.

PV-COF. 5,10,15,20-Tetrakis(4-aminophenyl)porphyrin (**1**, 50 mg, 0.074 mmol) and **2** (83.3 mg, 0.154 mmol) were introduced into a 35-mL microwave reaction vessel. A total volume of 15.6 mL of 4:1 mixture of ethanol and water was added, and the Zincke reaction was carried out under microwave irradiation at 90 °C for 2 h. The obtained solid was repeatedly washed with ethanol until the supernatant appeared clear. The solid product was dried overnight in a vacuum oven at 45 °C.

Zn-PV-COF. 80 mg of **PV-COF** was dispersed in 18 mL of anhydrous dichloromethane. In a separate flask, 600 mg of zinc acetate dihydrate ($Zn(OAc)_2 \cdot 2H_2O$) was dissolved in 9 mL of anhydrous methanol and added to the **PV-COF** suspension. The mixture was

refluxed at 50 °C for 24 h. After reaction, **Zn-PV-COF** solids were collected and washed extensively with methanol. Presence of Zn was confirmed by EDS.

Red-PV-COF. Reduction reaction was carried out entirely in a glovebox. 65 mg of cobaltocene $[Co(\eta_5C_5H_5)_2]$ was dissolved in 12 mL degassed MeCN and 50 mg of **PV-COF** was added to it. The mixture was shaken for 10 minutes and centrifuged. The pellet was repeatedly washed with degassed MeCN until the purple color of cobaltocene completely faded. **Red-PV-COF** was dried in a vacuum oven overnight.

Structural modeling. A crystal model was built with the use of *Biovia Materials Studio* software package. Based on the geometry of the building blocks, a model based on stacked square layers was created in the tetragonal space group *P-4*. Initially, the model was geometrically optimized using a universal forcefield based energy minimization, allowing cell parameters optimization. Following, the lattice parameters were further adjusted according to the position of the experimentally observed diffraction lines, and subsequent geometrical optimization cycles were run. Finally, four chloride anions per unit cell were introduced in the model, and their location in the cell was optimized with the use of the sorption module (locate task) of Materials Studio.

Bromate adsorption experiments. A stock solution of $NaBrO_3$ was prepared in water and diluted to various concentrations that were used to create a calibration curve. Quantification of $NaBrO_3$ was performed on an Agilent 1290 Infinity UHPLC coupled to a Bruker EVOQ triple quadrupole mass spectrometer. Agilent C-18 column (1.8-μm particles) was used with an inner diameter of 2.1 mm and length of 15 mm. Mobile phases consisted of 0.1 % formic acid for solvent A and 0.2 % formic acid in acetonitrile for solvent B. A sample volume of 10 μL for both calibration and unknown was injected onto the column. The samples were eluted from the column in isocratic mode for 3 minutes with solvent A. A 2-minute wash at 80 % B was used to keep the column sensitivity high and prevent carry-over, and a 2-minute equilibration at 0 % B was used to regenerate the column for next run. Column was attached to an Agilent UHPLC with a flow rate maintained at 400 μL min^{-1}. Selected/Multiple reaction monitoring (S/MRM) analyses were carried out on an EVOQ ESI-triple quadrupole mass spectrometer (Bruker) operated in negative ion mode. A calibration standard was used for creation of the transitions. Optimization of collision energy (CE) was performed for each transition tested. The final method for S/MRM included the following transitions and specifications: 128.85/112.9 (CE 21V), 128.85/97 (CE 31V), 126.85/110.9 (CE 21V), and 126.85/95.1 (CE 32V) where the precursors 128.85 and 126.85 correspond to the $[^{81}BrO_3]^-$ and $[^{79}BrO_3]^-$ respectively. The rest of the setting for the EVOQ triple quad mass spectrometer were as follows: spray voltage 4500 V, cone temperature 350 °C, cone gas 50 units, heated probe temperature 350 °C, probe gas 40 units, exhaust gas on, and nebulizer gas 40 units. Residual $NaBrO_3$ concentration was determined by means of the linear least square regression model after external calibration with calibration standards (n=5). Every calibration standard and sample was run in duplicates, with two blanks run before and after each sample run. The R^2 of the calibration curve was >0.99. Limit of detection was 0.1 μg L^{-1} and limits of quantification was 0.3 μg L^{-1}.

Bromate adsorption kinetics. To determine rate constants for bromate adsorption by various COFs, 10 mL of 50 µg L^{-1} BrO_3^- solution was transferred into a 20 mL glass vial and 5.0 mg of the COF was added to it. The mixture was sonicated for 10 s followed by stirring at 500 rpm. At various pre-determined time points, 1.0 mL was removed via syringe and passed through a cellulose syringe filter (Thermo Scientific Nalgene 0.45 µm SFCA). The filtrate was analyzed by EVOQ LC-MS liquid chromatography coupled to a triple quadrupole mass spectrometer. Concentrations of BrO_3^- in filtrates were determined by calculating area under the chromatogram curve and relating area to a calibration curve of known BrO_3^- standard solutions. Data were fitted to a pseudo-second order kinetics[1] model and the rate constants (k_{obs}) were determined from the linear fit according to the equation

$$t/q_t = t/q_e + 1/(k_{obs} \cdot q_e^2),$$

where q_t is the quantity of BrO_3^- adsorbed at time t (min), q_e is the adsorbed amount at equilibrium, and k_{obs} is the second-order rate constant (g mg^{-1} min^{-1}).

Bromate adsorption isotherms. To determine maximum quantity of bromate adsorbed by each of the COFs, 2 mL of BrO_3^- solutions ranging in concentration from 12.5 to 300.0 mg L^{-1} was prepared. Next, 1 mg of a COF was added, the mixture was sonicated for 10 s and stirred at 500 rpm for 30 min. Finally, 1.0 mL was removed via syringe and passed through a cellulose syringe filter (Thermo Scientific Nalgene 0.45 µm SFCA). The filtrate was analyzed by EVOQ LC-MS. Concentrations of BrO_3^- in filtrates were determined by calculating area under the chromatogram curve and relating area to a calibration curve of known BrO_3^- standard solutions. Data were fitted to the Langmuir adsorption model[2]

$$q_e = q_m \cdot b \cdot C_e/(1+b \cdot C_e),$$

where q_e (mg g^{-1}) is the amount of BrO_3^- adsorbed at equilibrium, C_e (mg L^{-1}) is the equilibrium solute concentration remaining in solution when q_e is achieved, q_m is the maximum adsorption capacity corresponding to complete monolayer coverage, and b is a constant (L mg^{-1}).

Regeneration experiments. BrO_3^- was desorbed from **PV-COF** using a previously described method with slight modifications.[3] Briefly, in the first adsorption cycle 5 mg of **PV-COF** was added to 10 mL of 50 µg L^{-1} BrO_3^- and the mixture was stirred at 500 rpm for 30 min. The COF was collected by centrifugation and washed three times with 15 mL of 50 mM NaOH and with 10 mM HCl until the supernatant was neutralized. After a final water wash, **PV-COF** was dried overnight in a vacuum oven at 45 °C and reused for subsequent cycles. In addition to the industry-relevant BrO_3^- concentration of 50 µg L^{-1}, the material was also reused for adsorption of 50 mg L^{-1}, using the same protocol for desorption and re-adsorption.

Modeling interaction between BrO_3^- and PV-COF. An energy minimization including a portion of the central porphyrin moiety and the bromate anions was performed with the density functional based thigh binding (DFTB+) method, implemented in *Materials Studio* software. The halorg Slater-Koster library was used,[4] with energy convergence tolerance of 0.05 kcal mol^{-1}. For the location of the bromate ions in the periodic porous **PV-COF** structure, a Monte Carlo based simulation was completed with the use of the *Sorption*

module of *Materials Studio*, employing a Dreiding force field. Four bromate anions were included per unit cell.

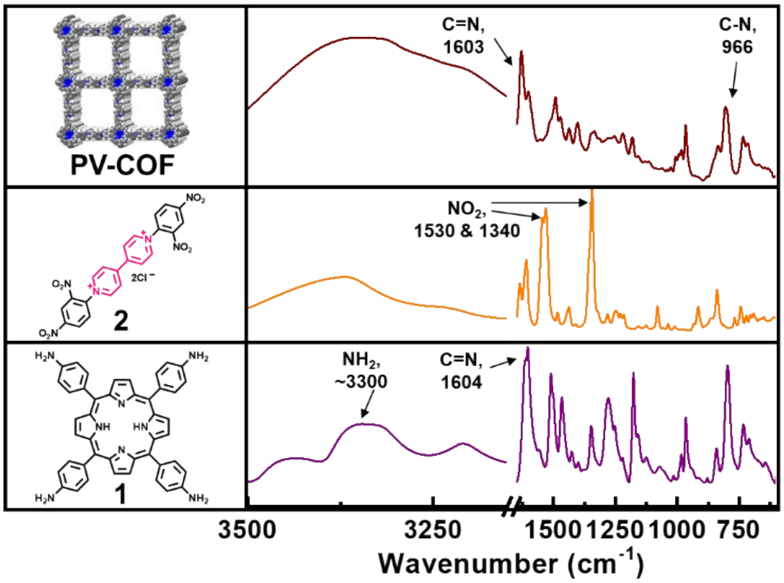

Fig. S1. FT-IR spectra of **PV-COF** (top) and the corresponding monomers, 5,10,15,20-tetrakis(4-aminophenyl)porphyrin (**1**, bottom) and 1,1'-bis(2,4-dinitrophenyl)-[4,4'-bipyridine]-1,1'-diium dichloride (**2**, middle).

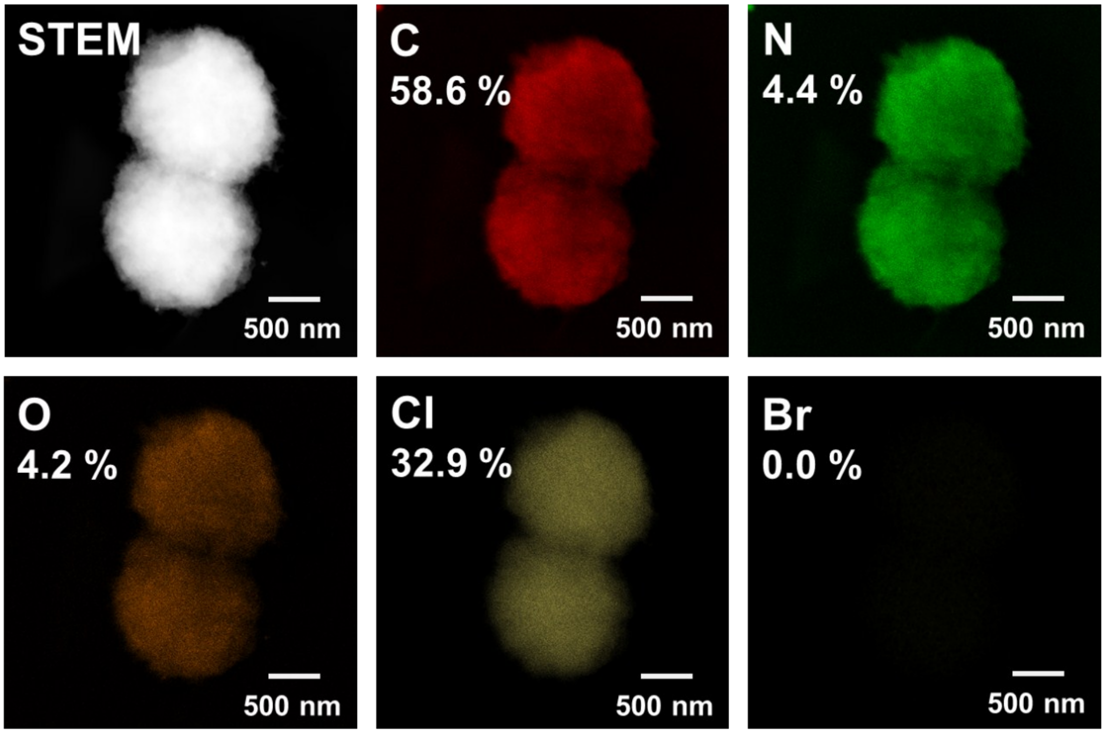

Fig. S2. STEM image of **PV-COF** and EDS elemental mapping showing an even distribution of constituent elements C, N and Cl.

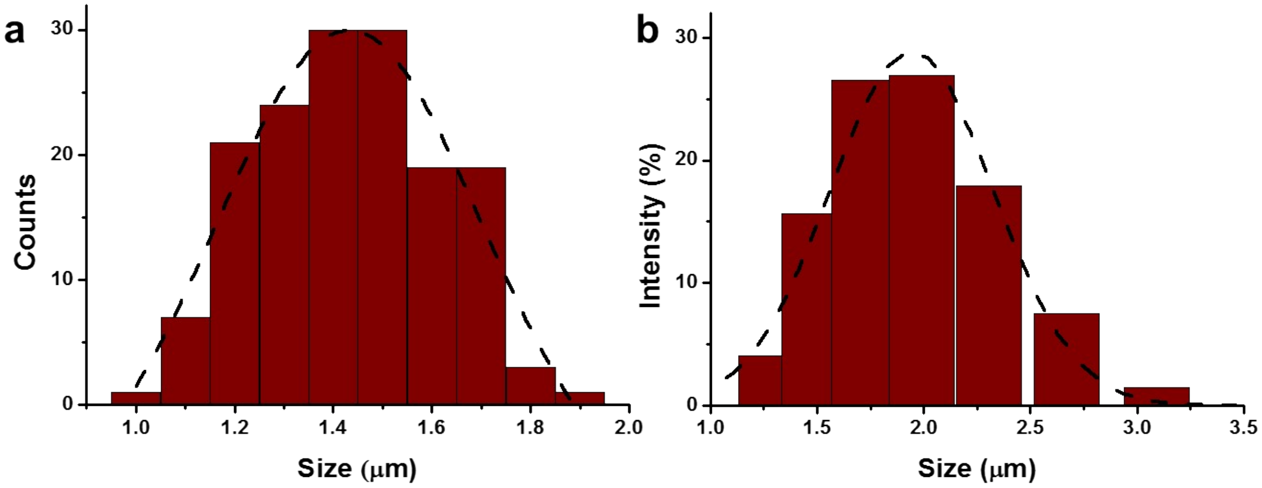

Fig. S3. Size distributions of **PV-COF** in TEM (a, d_{avg} = 1.4 μm) and DLS (b, d_{avg} = 1.8 μm).

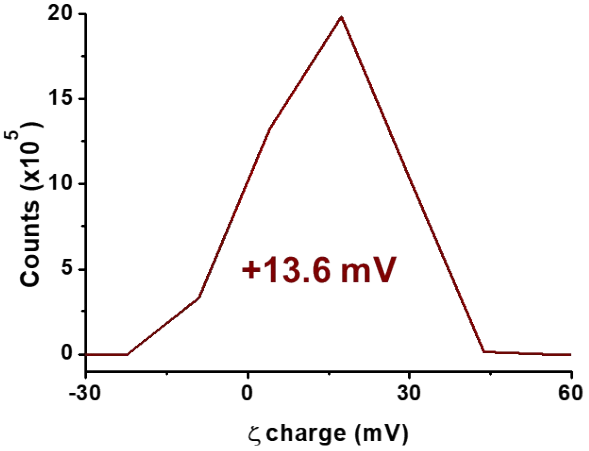

Fig. S4. ζ-potential measurement for **PV-COF** shows an average of +13.6 mV.

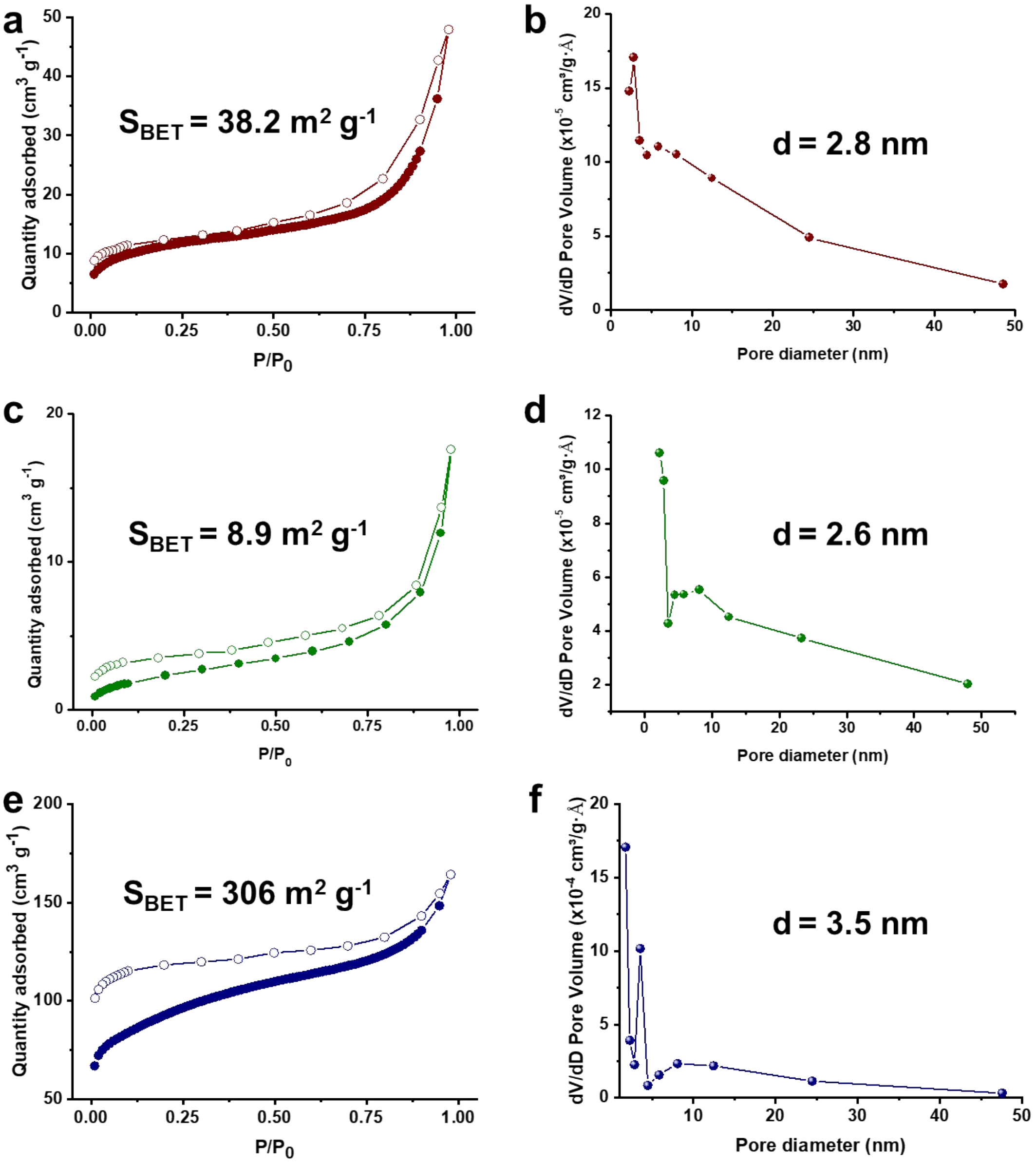

Fig. S5. N_2 adsorption isotherms and BJH pore size distributions for **PV-COF** (a–b), **Zn-PV-COF** (c–d), and **Red-PV-COF** (e–f).

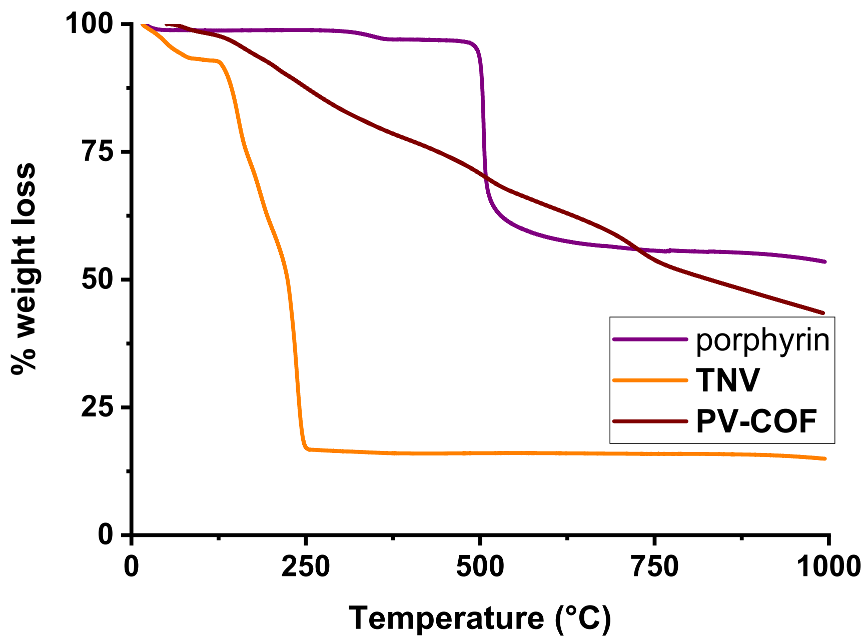

Fig. S6. Thermogravimetric analyses of **PV-COF** (brown), TNV (orange) and starting porphyrin monomer (purple).

Table S1. Lattice parameters and fractional atomic coordinates of **PV-COF**.

Space group	*P-4*		
a (Å)	25.25		
c (Å)	4.03		
Atom	Fractional coordinates		
	x	*y*	*z*
N1	0.05548	0.05625	-0.00673
C2	0.04693	0.10834	-0.06539
C3	0.09321	0.13269	-0.16779
C4	0.22237	0.03993	0.17926
C5	0.27740	0.04070	0.17699
C6	0.38875	0.04461	-0.10182
C7	0.44374	0.04483	-0.10168
H8	0.17252	0.10274	-0.21976
H9	0.20174	0.06991	0.31708
H10	0.29831	0.07094	0.31529
H11	0.36775	0.07890	-0.19604
H12	0.46477	0.07899	-0.19694
C13	0.13511	0.00094	0.00019
C14	0.19427	0.00135	0.00048
C15	0.30534	0.00158	0.00516
N16	0.36176	0.00135	0.01020

C17	0.47102	0.00038	0.01569
C18	0.44267	0.95655	0.13160
C19	0.38772	0.95774	0.12619
C20	0.27791	0.96245	-0.16987
C21	0.22291	0.96303	-0.17707
C22	0.89231	0.95157	-0.06507
C23	0.86859	0.90497	-0.16741
H24	0.10036	0.17388	-0.22067
H25	0.46286	0.92196	0.22793
H26	0.36590	0.92376	0.21707
H27	0.29922	0.93215	-0.30515
H28	0.20270	0.93291	-0.31626
Cl	0.59185	0.16645	0.68279

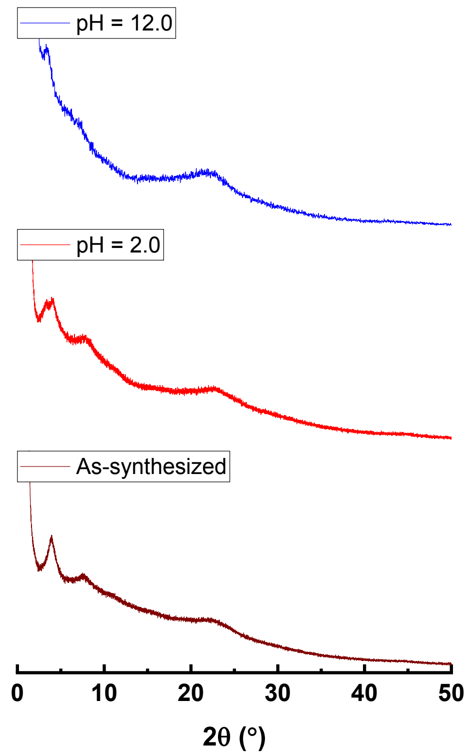

Fig. S7. PXRD spectra of as-synthesized **PV-COF**, **PV-COF** exposed to acidic (pH = 2.0) and basic solutions (pH = 12.0).

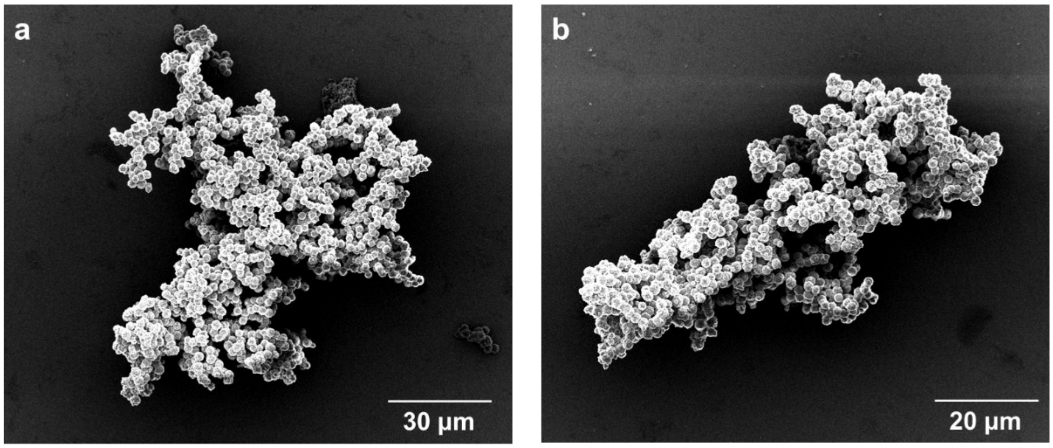

Fig. S8. SEM images of **PV-COF** acquired after exposure to pH = 2.0 (a) and pH = 12.0 for 24 h at room temperature.

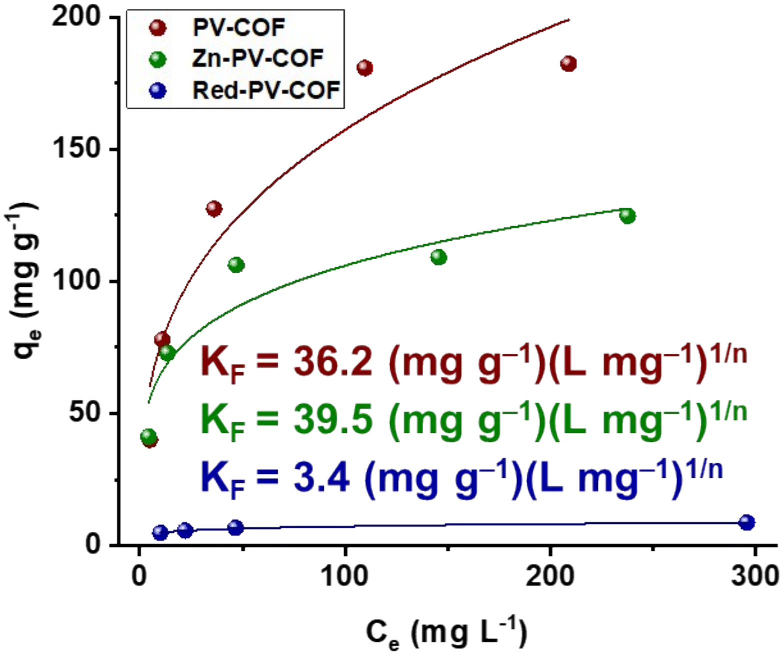

Fig. S9 Isotherm fitting to the Freundlich model for **PV-COF**, **Zn-PV-COF** and **Red-PV-COF** shows poorer agreement with the data than the Langmuir model.

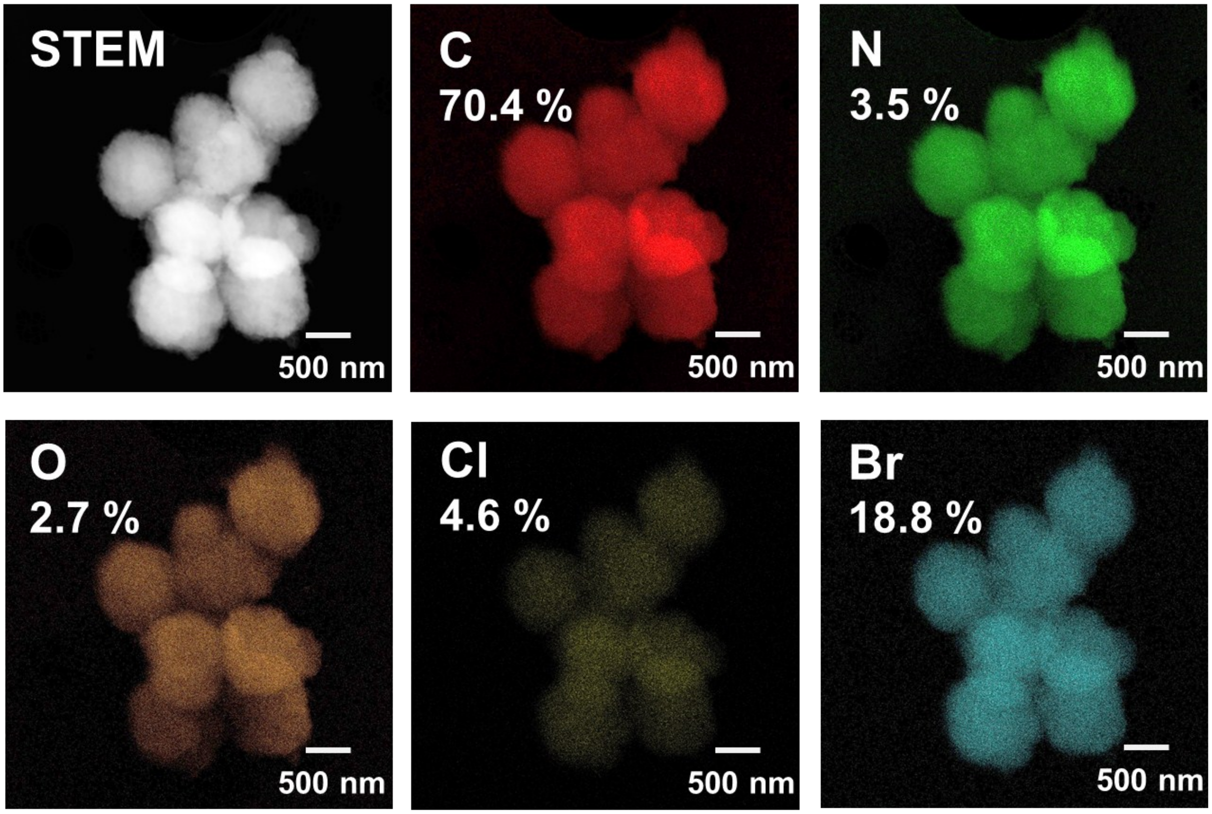

Fig. S10. STEM image of **PV-COF** following BrO_3^- adsorption and EDS elemental mapping showing an even distribution of constituent elements C, N and Cl along with adsorbed Br from BrO_3^-.

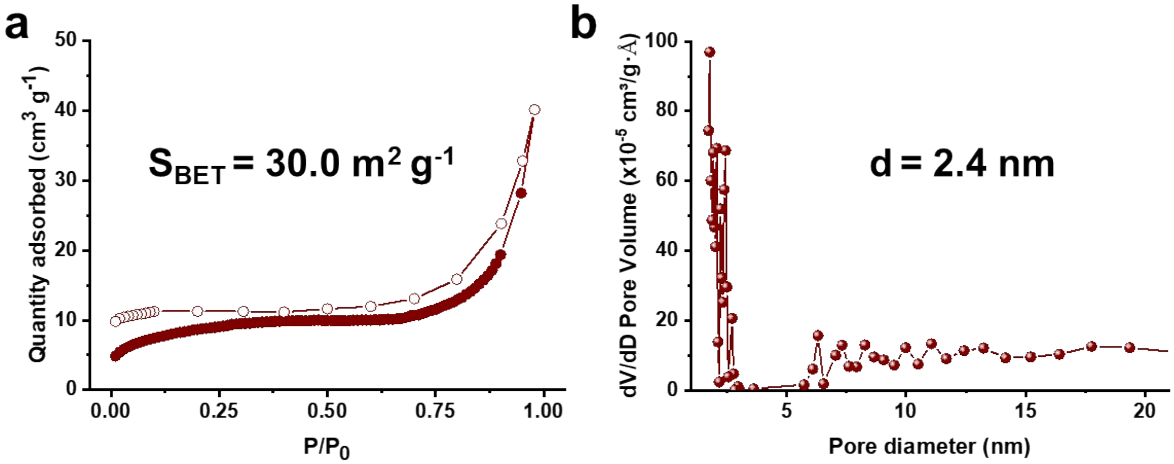

Fig. S11. Gas adsorption experiments of **PV-COF** after incubation in $NaBrO_3$ solution. N_2 adsorption isotherm (a) and pore size distribution (b).

Table S2. Bromate adsorbents reported in the literature and their corresponding rate constants and maximum adsorption capacities.

Material	Ref.	Q_{max} (mg g^{-1})	k (g mg^{-1} min^{-1})
**PV-COF**	**This work**	**203.80**	**191.45**
Soil	5	0.00462	41
Carbon-immobilized nano zero-valent iron	6	31.82	4.19
Zn-Al calcined layered double hydroxide	7	n/a	1.577
Chitosan polymers	3	43.36	0.918
Amorphous aluminum hydroxide	8	n/a	0.762
Nano-Al_2O_3	9	6.66	0.578
Granular ferric hydroxide (GFH)	10	17.86	0.519
Cationic surfactant-modified powdered activated carbon	11	35.8	0.448
Fe-pillared bentonite	12	17.42	0.35
Bamboo-based activated carbon	13	16.3	0.264
Fe-CNT nanocomposite	14	72.12	0.1989
Ordered mesoporous carbon	15	24.378	0.0970
Cationic surfactant-modified granular activated carbon	16	38.02	0.0313
Various commercial activated carbons	17	90.9	0.000517
Modified granular activated carbon	18	46.79	0.0268
Agricultural waste-based activated carbon	19	25.64	0.0215
Ordered mesoporous Mg-Al layered double hydroxides	20	59.34	0.017
β-FeOOH-coupled activated carbon	21	87.92	0.0138
Resin impregnated with hydrated ferric oxide	22	292.81	0.0085
Fruit-based powdered activated carbon	23	99.6	0.00817
Corncobs	24	101.01	0.0011
Thiol-functionalized MOF	25	59.6	0.000417
Resin Amberlite IRA-400	26	1.99	0.000342
Nano-iron hydroxide impregnated granular activated carbon (Fe-GAC)	27	45.27	0.0000008327
Nano-iron hydroxide impregnated granular activated carbon	28	42.74	0.0000007762
Chitosan-based molecularly imprinted polymer sol–gel ion-exchange double hydrous oxide cryogel	29	0.200	0.00000033

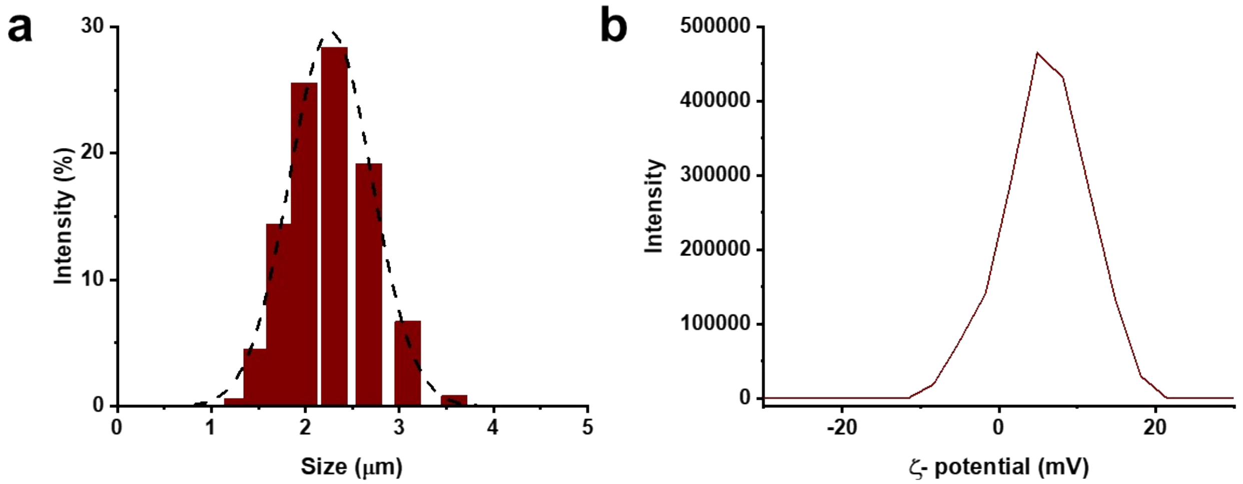

Figure S12. a) Size distribution of **PV-COF** in DLS after adsorption of bromate (d_{avg} = 2.2 μm); b) ζ-potential of **PV-COF** post bromate adsorption ($ζ_{avg}$ = 5.2 mV).

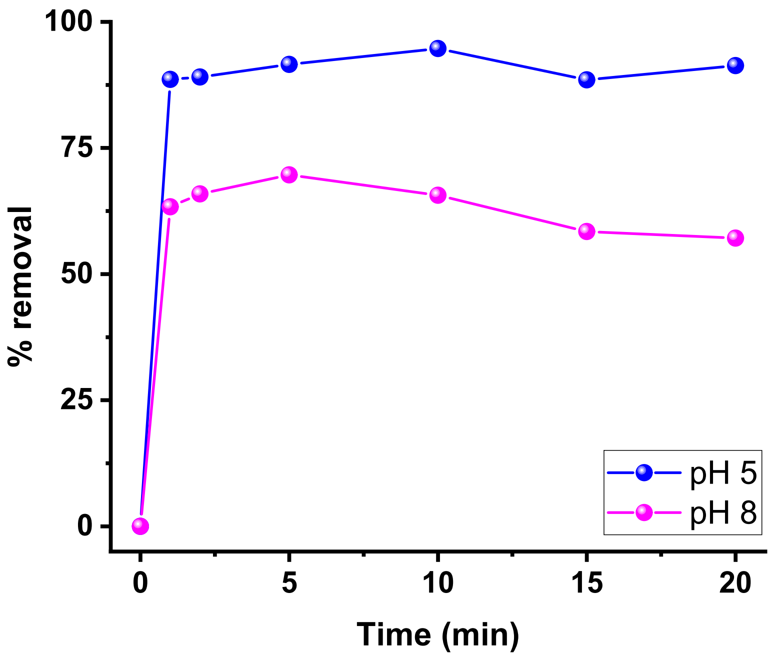

Fig. S13. % removal of BrO_3^{-1} by **PV-COF** with 50 μg L^{-1} initial concentration of BrO_3^- in either acidified (pH = 5) or basified (pH = 8) solutions at different time points.

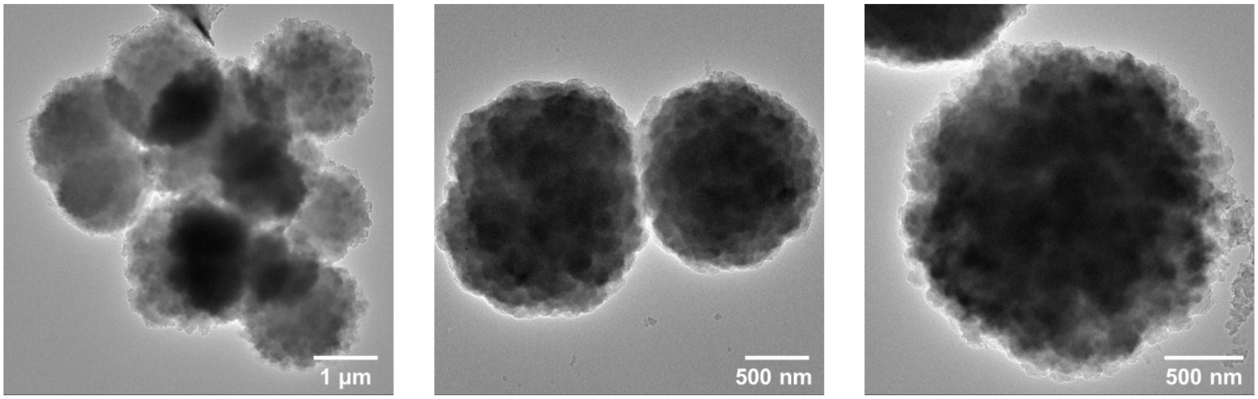

Fig. S14. TEM images of **Zn-PV-COF**, which exhibits no change in morphology following metalation of porphyrin subunits.

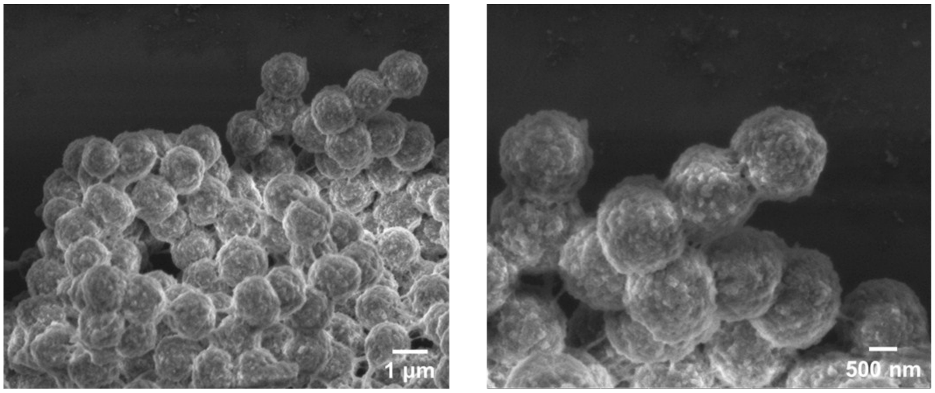

Fig. S15. SEM images of **Red-PV-COF**, which exhibits no change in morphology following metalation of porphyrin subunits.

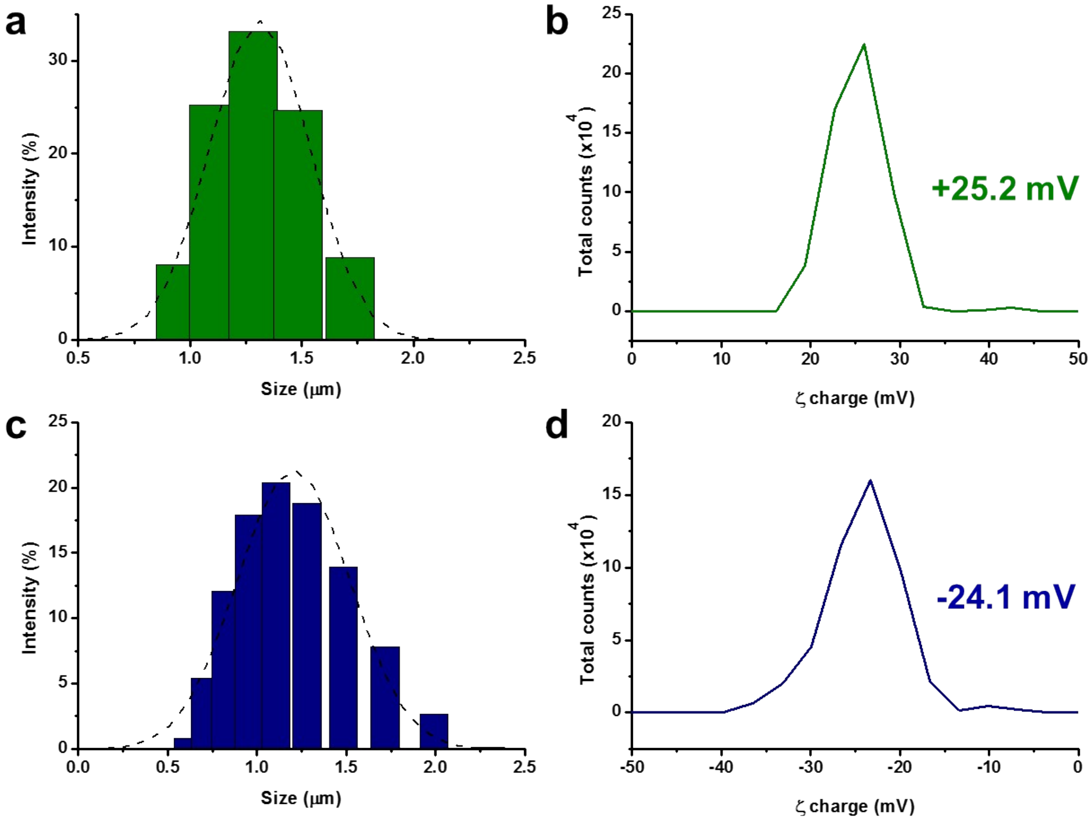

Fig. S16. a) Size distribution of **Zn-PV-COF** (d_{avg} = 1.4 μm); b) ζ-potential distribution of **Zn-PV-COF**; c) size distribution of **Red-PV-COF** (d_{avg} = 1.2 μm); d) ζ-potential distribution of **Red-PV-COF**.

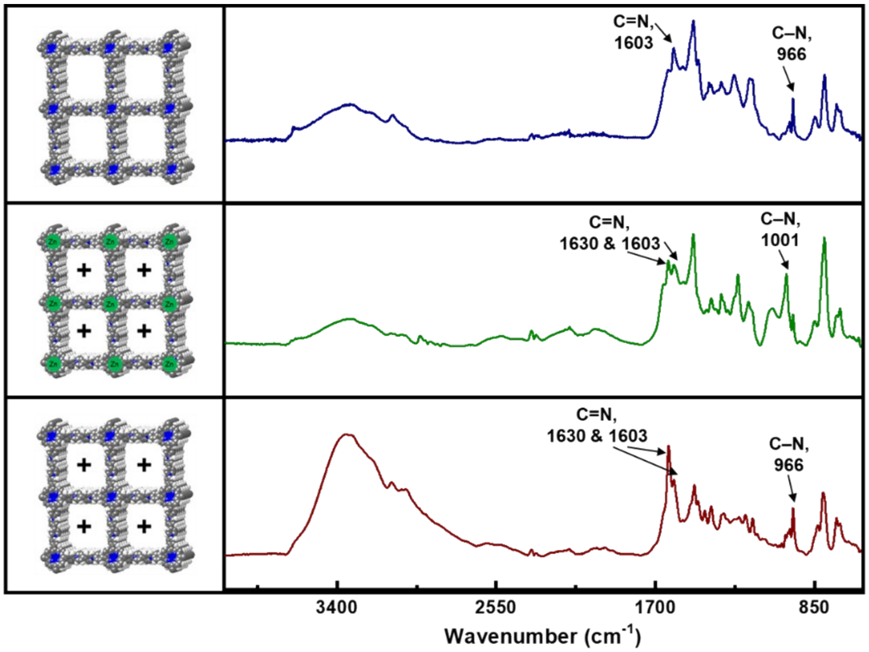

Fig. S17. FT-IR spectra of **PV-COF** (brown), Zn-metallated **Zn-PV-COF** (green), and reduced **Red-PV-COF** (blue).

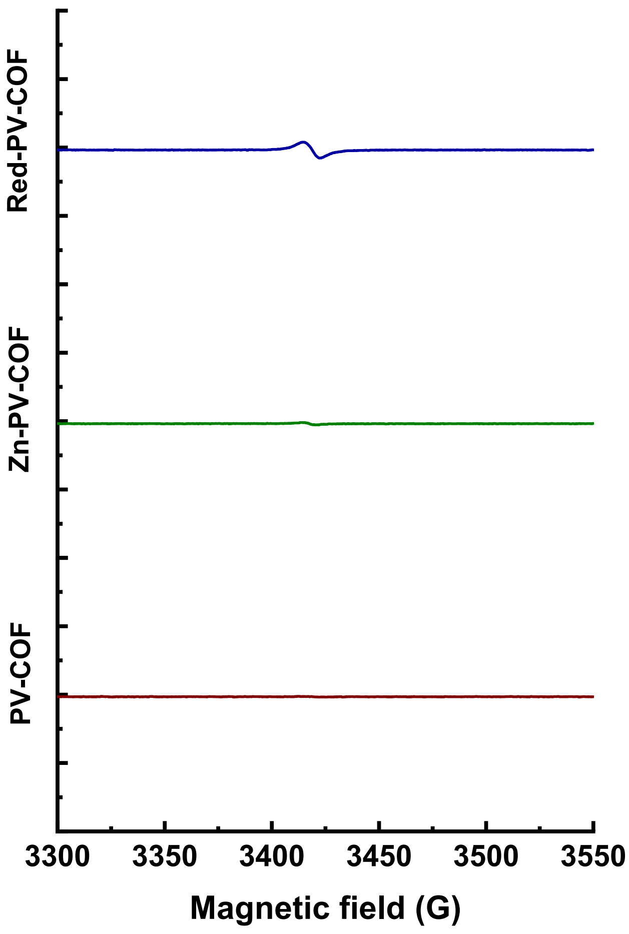

Fig. S18. EPR spectra of **PV-COF** (brown), **Zn-PV-COF** (green), and **Red-PV-COF** (blue).

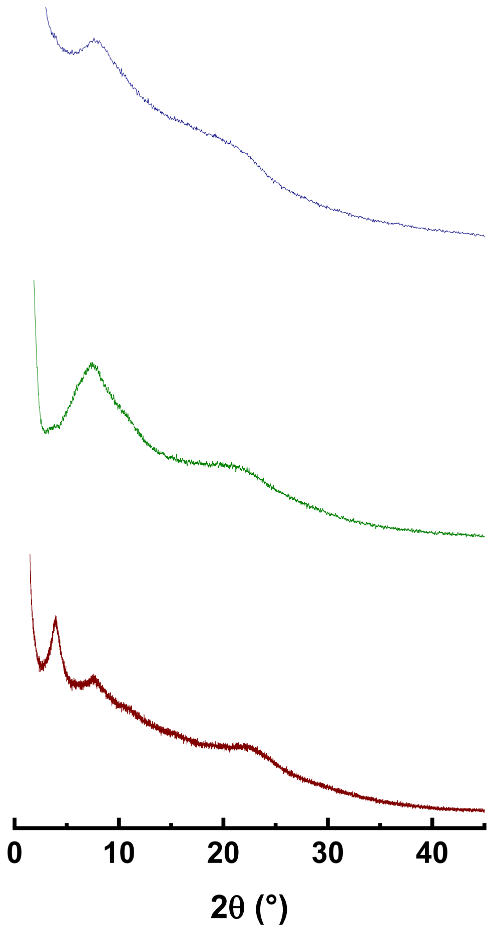

Fig. S19. PXRD spectra of **PV-COF** (bottom, brown), **Zn-PV-COF** (middle, green) and **Red-PV-COF** (top, blue).

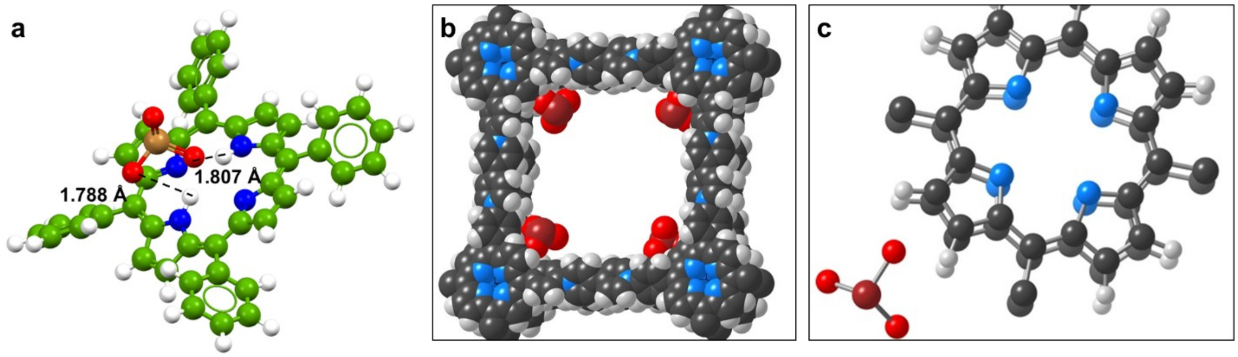

Fig. S20. Simulation of porphyrin – bromate interaction. a) Interaction between the bromate anion and an individual porphyrin ring indicates formation of hydrogen bonds between the O of bromate and H of pyrrole rings in porphyrin. Calculated hydrogen bonds are 1.788 and 1.807 Å long. b) In a periodic **PV-COF** structure, bromate molecules interact with pyrrole rings of the porphyrin core to form hydrogen bonds. The image specified interaction of the bromate anions in the pores of **PV-COF**. c) Detailed view of one of the sorption sites.

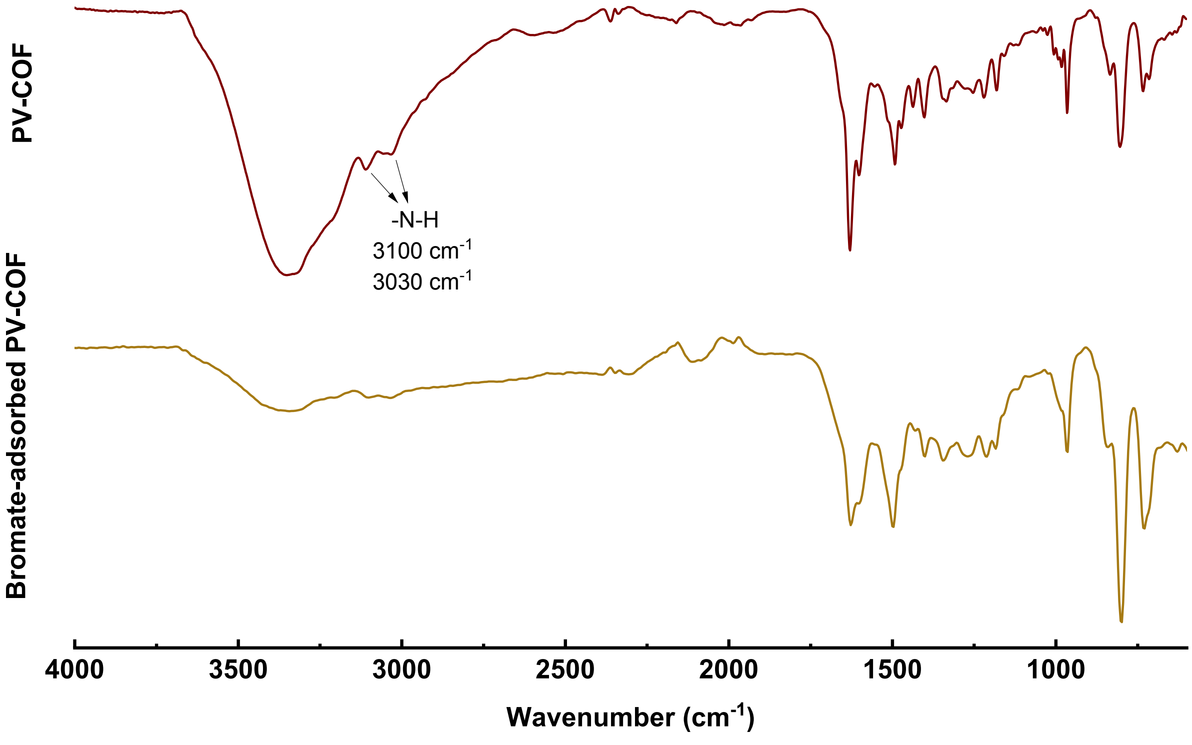

Figure **S21.** FT-IR spectra of as-synthesized **PV-COF** (brown) and bromate-adsorbed **PV-COF** (gold).

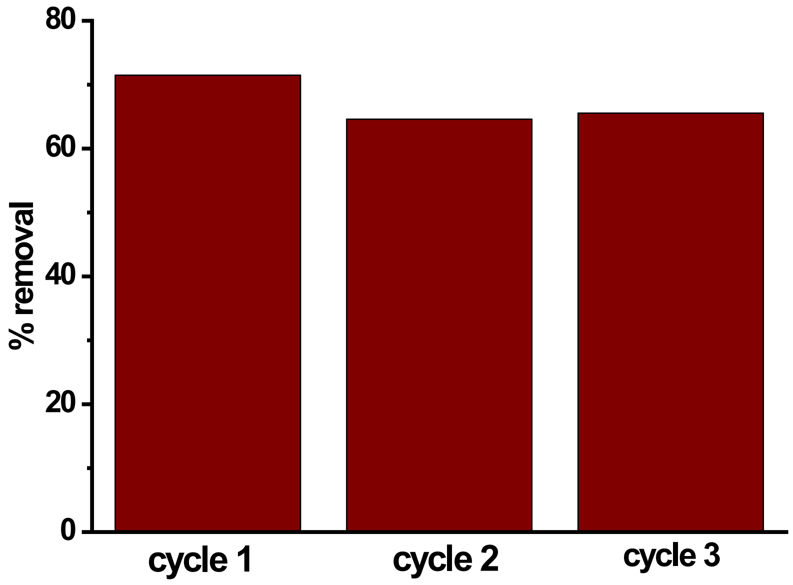

Fig. S22. Percentages of 50 mg L^{-1} BrO_3^- adsorbed by **PV-COF** after three consecutive regenerations.

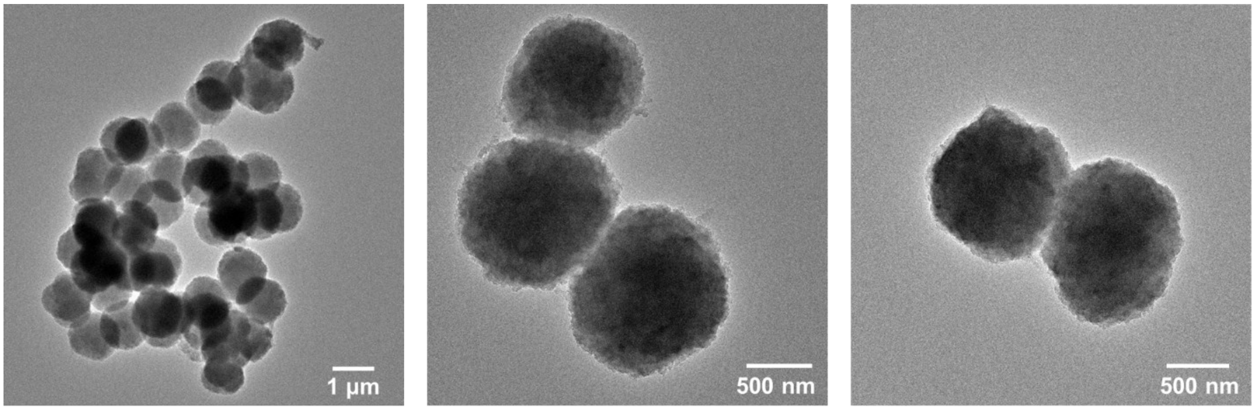

Fig. S23. TEM images recorded after the third **PV-COF** regeneration.

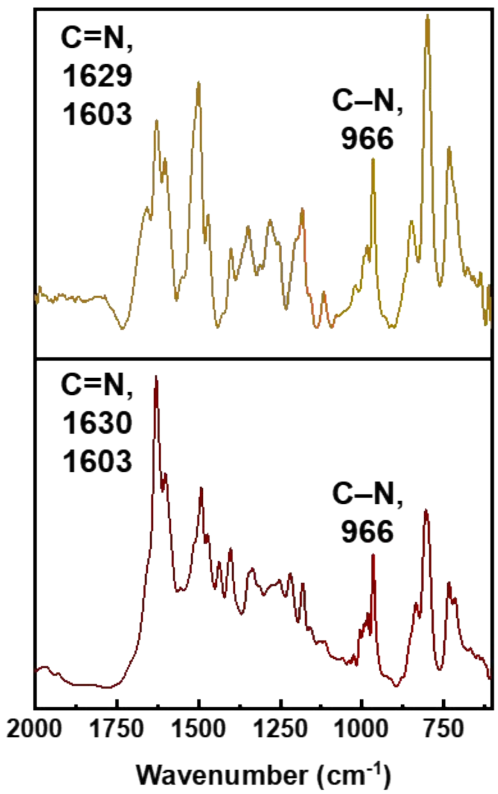

Fig. S24. FT-IR spectra of as-synthesized **PV-COF** (brown) and regenerated **PV-COF** (gold).

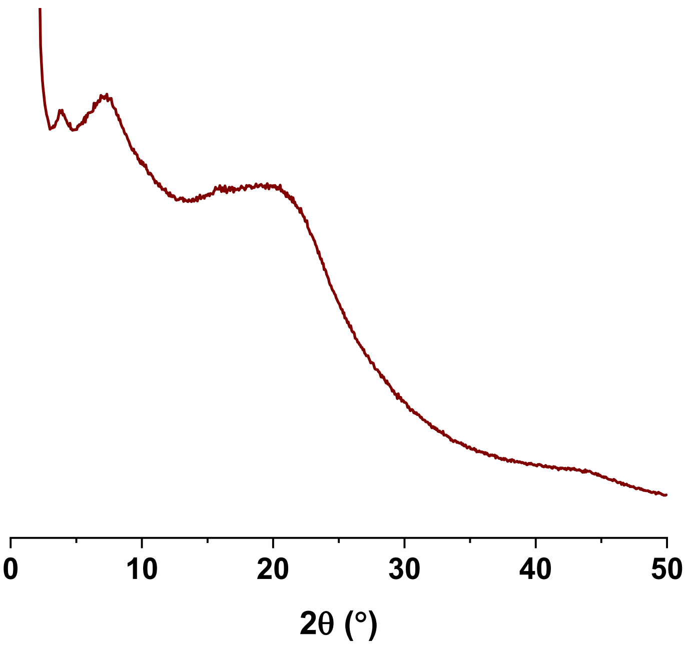

Fig. S25. PXRD pattern of PV-COF after three cycles of regeneration.

Flow experiments. A 1-mL syringe was equipped with three layers of filter paper at the bottom, charged with ~50 mg of **PV-COF** and capped with a syringe filter (Fig. S22). Bromate solution (50 µg L^{-1}) w[illegible] syringe pump (New Era Pump Systems, model NE-300) and [illegible] The filtrate was analyzed with the same method on HPLC-M[illegible] was regenerated by passing through the same syringe a so[illegible]llowed by a solution of HCl (10 mM) before being used in the [illegible] for a total of three adsorption cycles.

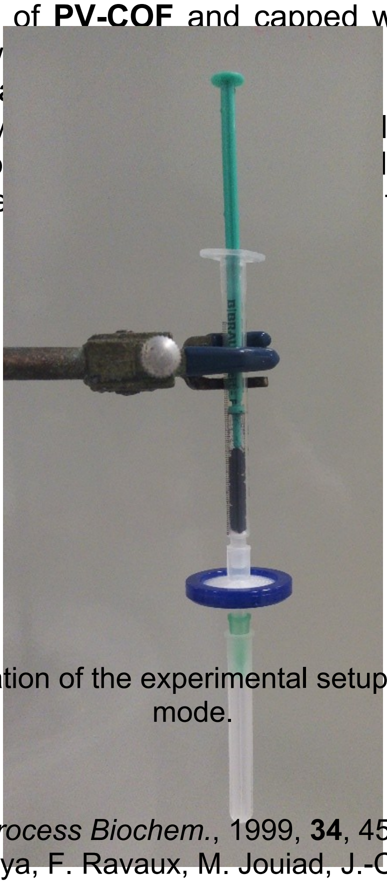

Fig. S26. Schematic representation of the experimental setup for BrO_3^- adsorption in the flow mode.

References

1 Y.-S. Ho and G. McKay, *Process Biochem.*, 1999, **34**, 451–465.
2 D. Shetty, I. Jahovic, J. Raya, F. Ravaux, M. Jouiad, J.-C. Olsen and A. Trabolsi, *J. Mater. Chem. A*, 2017, **5**, 62–66.
3 J. Liu, W. Shi, Y. Liu, W. Ou-Yang and R. Zhao, *Water Sci. Technol. Water Supply*, 2017, **17**, 1062–1069.
4 T. Kubař, Z. Bodrog, M. Gaus, C. Köhler, B. Aradi, T. Frauenheim and M. Elstner, *J. Chem. Theory Comput.*, 2013, **9**, 2939–2949.
5 A. Jia, C. Wu, W. Hu and C. Hu, *Clean - Soil, Air, Water*, 2015, **43**, 1072–1077.
6 C. hua Xu, S. Lin, X. hong Wang, Y. ming Chen, L. jia Zhu and Z. hua Wang, *J. Taiwan Inst. Chem. Eng.*, 2014, **45**, 3000–3006.
7 Y. Zhang and X. Li, *J. Environ. Eng.*, 2014, **140**, 04014018.
8 R. Chitrakar, K. Mizobuchi, A. Sonoda and T. Hirotsu, *Ind. Eng. Chem. Res.*, 2010, **49**, 8726–8732.
9 A. Bhatnagar and M. Sillanpää, *Sep. Sci. Technol.*, 2012, **47**, 89–95.
10 A. Bhatnagar, Y. H. Choi, Y. J. Yoon, Y. Shin, B. H. Jeon and J. W. Kang, *J. Hazard. Mater.*, 2009, **170**, 134–140.
11 W. Farooq, H.-J. Hong, E. J. Kim and J.-W. Yang, *Sep. Sci. Technol.*, 2012, **47**, 1906–1912.
12 S. He, D. Zhang, L. Gu, S. Zhang and X. Yu, *Environ. Technol. (United Kingdom)*, 2012, **33**, 2337–2344.
13 H.-W. Chen, Y. H. Chuang, C.-F. Hsu and W.-J. Huang, *J. Environ. Sci. Heal. Part A*, 2017, **52**, 1055–1062.
14 I. H. Alsohaimi, M. Ali Khan, Z. A. Alothman, M. R. Khan, M. Kumar and A. M. Al mahri, *J. Ind. Eng. Chem.*, 2015, **26**, 218–225.
15 C. Xu, X. Wang, X. Shi, S. Lin, L. Zhu and Y. Chen, *Environ. Technol.*, 2014, **35**, 984–

992.
16 W. Chen, Z. Y. Zhang, Q. Li and H. Y. Wang, *Chem. Eng. J.*, 2012, **203**, 319–325.
17 W. J. Huang and Y. L. Cheng, *Sep. Purif. Technol.*, 2008, **59**, 101–107.
18 H. Yan, X. Du, P. Li, S. Yu and Y. Tang, *Ozone Sci. Eng.*, 2015, **37**, 357–370.
19 M. Naushad, M. R. Khan, Z. A. ALOthman, I. AlSohaimi, F. Rodriguez-Reinoso, T. M. Turki and R. Ali, *Environ. Sci. Pollut. Res.*, 2015, **22**, 15853–15865.
20 H. Ji, W. Wu, F. Li, X. Yu, J. Fu and L. Jia, *J. Hazard. Mater.*, 2017, **334**, 212–222.
21 D. Han, Z. Zhao, Z. Xu, Y. Li, P. Zhang and X. Guo, *J. Chem. Eng. Data*, 2018, **63**, 2243–2251.
22 S. Li, Q. Yang, Y. Zhong, F. Chen, T. Xie, F. Yao, J. Sun, C. Jiang, X. Li and G. Zeng, *J. Chem. Eng. Data*, 2016, **61**, 1305–1312.
23 L. Wang, J. Zhang, J. Liu, H. He, M. Yang, J. Yu, Z. Ma and F. Jiang, *J. Environ. Sci.*, 2010, **22**, 1846–1853.
24 Y. Cui, Z. Hu, J. Chen and Z. Yan, *Desalin. Water Treat.*, 2011, **28**, 338–344.
25 P. Han and Y. Xia, *J. Environ. Chem. Eng.*, 2018, **6**, 3384–3391.
26 M. Naushad, M. R. Khan, Z. A. ALOthman and M. R. Awual, *Desalin. Water Treat.*, 2016, **57**, 5781–5788.
27 J. hong Xu, N. yun Gao, D. ye Zhao, W. xian Zhang, Q. kun Xu and A. hong Xiao, *Chem. Eng. J.*, 2015, **275**, 189–197.
28 J. H. Xu, N. Y. Gao, D. Y. Zhao, D. Q. Yin, H. Zhang, Y. Q. Gao and W. Shi, *Sep. Purif. Technol.*, 2015, **147**, 9–16.
29 S. Hajizadeh, H. Kirsebom, I. Y. Galaev and B. Mattiasson, *J. Sep. Sci.*, 2010, **33**, 1752–1759.
